# Ultrasound-assisted synthesis of 2,4-thiazolidinedione and rhodanine derivatives catalyzed by task-specific ionic liquid: [TMG][Lac]

**DOI:** 10.1186/2191-2858-3-2

**Published:** 2013-03-03

**Authors:** Suresh, Jagir Singh Sandhu

**Affiliations:** 1 Department of Chemistry, Punjabi University, Patiala, Punjab, 147 002, India

**Keywords:** 2,4-thiazolidinedione, Rhodanine, Knoevenagel condensation, Recyclability, Task-specific ionic liquid

## Abstract

**Background:**

Synthesized arylidene derivatives of rhodanine and 2,4-thiazolidiendione have potent pharmacological activities, and these are also key substrates for the preparation of clinically used antidiabetics.

**Findings:**

Some 1,1,3,3-tetramethylguanidine-based task-specific ionic liquids (TSILs) **1a**-**1e** were prepared and employed to the catalyzed solvent-free Knoevenagel condensation of 2,4-thiazolidinedione **3a** and rhodanine **3b** with a variety of aldehydes.

**Conclusions:**

Best results were obtained with 1,1,3,3-tetramethylguanidine lactate ([TMG][Lac]) **1c**. The TSIL used can be easily recovered and recycled, yielding products **4**–**5** in excellent yields under ultrasonic environment without the formation of any side products or toxic waste.

## Findings

### Background

**Figure 1:**
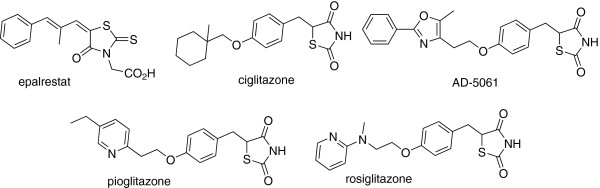
**Clinically used molecules having 5-arylidene rhodanines and 2,4-thiazolidenediones.**

2,4-thiazolidenedione (TZD) is an attractive scaffold because of its prestigious position in medicinal chemistry as this unit is responsible for numerous pharmacological and biological activities, e.g., antidiabetic [1,2], antidiarrheal [3], anticonvulsant [4], antimicrobial [5], antihistaminic [6], anticancer [7], anti-HIV [8], 15-hydroxyprostaglandin dehydrogenase inhibitors [9], and anti-ischemic [10]. The position of these molecules seems to be most significant as they are a subset of commercially employed non-insulin-dependent diabetes mellitus and insulin-sensitizing agents (Figure 1) such as rosiglitazone, epalrestat, ciglitazone, AD-5061, pioglitazone, and so on.

Several methods have been developed for their condensation with aldehydes as this is a crucial step in the production of the above clinically used drugs (Figure 1), and thus, this condensation is of commercial value. To achieve this end, several production protocols are reported employing several catalysts used such as amines [11], amine derivatives [12], amines salts [13], baker's yeast [14], sodium acetate [15-17], glycine [18,19], KF-Al_2_O_3_[20], and ionic liquids [21-25]. Several of these existing protocols for achieving this step have drawbacks like having long reaction times and low yields and leaving toxic residues on aqueous work-up; amine use is also now found to be carcinogenic. In addition, ionic liquids used earlier to accelerate this reaction are not environmentally benign because their preparation involves the harmful chemical intake, that is, volatile solvent and corrosive/toxic reagents [21-25]. Hence, a facile efficient process is still desirable. In search of greener chemical process development, ionic liquids are finding a prominent place [26-30]; herein, we report the first-ever sonically enhanced procedure using the environmentally benign ionic liquid 1,1,3,3-tetramethylguanidine lactate ([TMG][Lac]).

This ionic liquid based on TMGs is stable and easy to prepare in the laboratory, e.g., [TMG][Lac] **1c** is prepared by neutralizing TMG with lactic acid [31]. [TMG][Lac] has already been reported to accelerate important reactions such as Henry reactions, hydrogenation of olefins, hydrogenolysis of glycerol, aldol reaction, and so on [32-38]. Our own interest in ionic liquids (ILs) and in green synthetic transformations [29,30] prompted us to explore the catalytic activities of TMG-based ILs in the synthesis of 2-TZD and rhodanine derivatives. On top of all these factors, sonication is finding extensive use as it saves time and is environmentally friendly [39-41].

### Results and discussion

In our initial pilot experiments, ionic liquids **1a**-**1e** based on TMGs were used to catalyze condensation of 4-methoxybenzaldehyde **2a** with 2,4-thiazolidinedione **3a** (Table 1). The best results were obtained when the reaction was catalyzed by task-specific ionic liquid (TSIL) [TMG][Lac] **1c** under ultrasound irradiations at 80°C for 20 min (Table 1, entry 3) among the ILs **1a**-**1e**. The amount of catalyst **1c** was also established as 20 mol% by carrying out reactions of **2a** with **3a** using different amounts of catalyst (5 to 50 mol%).

**Table 1:** **Synthesis of** (***Z***)-**5**-(**4**-**methoxybenzylidene**)**thiazolidine**-**2**,**4**-**dione 4a via Knoevenagel condensation of 2a with 3a employing different ILs as catalyst**

		
**Entry**	**ILs (20 mol%)**	**Time (min)**	**Yield (%)**^**a**^
1	[TMG][CH_3_COO^−^]: **1a**	50	92
2	[TMG][CF_3_COO^−^]: **1b**	120	85
3	[TMG][CH_3_CHOHCOO^−^]: **1c**	20	95
4	[TMG][CH_3_CH_2_COO^−^]: **1d**	75	91
5	[TMG][(CH_3_)_2_CH_2_COO^−^]: **1e**	40	94

The reaction conditions for (*Z*)-5-(4-methoxybenzylidene)thiazolidine-2,4-dione **4a** are as follows: **2a** (1 mmol), **3a** (1 mmol), and catalysts (20 mol%) were irradiated under ultrasound irradiations at 80°C. The product was characterized by spectral techniques like IR, ^1^H NMR, ^13^C NMR, and mass spectra. ^a^Isolated yields after recrystallization.

To check the effect of catalyst, temperature, and ultrasound irradiations, a set of reactions was performed using different reaction conditions such as heating alone (Table 2, entry 2), ultrasound alone (Table 2, entries 6 and 7), heating along with ultrasound irradiations (Table 2, entries 5 to 10), and in the absence of both (Table 2, entry 1). Conclusively, catalyst, temperature, and ultrasound irradiations are all equally important to accelerate the Knoevenagel condensation of arylaldehydes **2** and 2,4-thiazolidenedione/rhodanine **3** to afford 5-arylidene-2,4-thiazolidinediones and 5-arylidenerhodanines **4**–**5** with excellent yields (Table 2, entry 9).

**Table 2:** Condensation of 2a and 3a in the presence of different reaction conditions

**Entry**	**Catalyst**	**Time (min)**	**Yield**^**a**^
1	No catalyst + No Heating + No US	180	-
2	No catalyst + Heating (50°C)	180	-
3	Catalyst (20 mol%) + Heating (50°C)	60	<55
4	Catalyst (20 mol%) + Heating (80°C)	60	68
5	No catalyst + Heating (50°C) + US	120	<10
6	Catalyst (20 mol%) + No Heating + US	50	74
7	Catalyst (50 mol%) + No Heating + US	50	72
8	Catalyst (20 mol%) + Heating (50°C) + US	40	80
9	Catalyst (20 mol%) + Heating (80°C) + US	20	95
10	Catalyst (20 mol%) + Heating (100°C) + US	30	92

The different reaction conditions are as follows: **1a** (1 mmol), **2a** (1 mmol), and catalysts were irradiated under ultrasound irradiations (US) at different temperatures. ^a^Isolated yield.

**Scheme 1:**
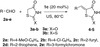
Knoevenagel reaction of 2 and 3 catalyzed by 1c.

Further, a variety of carbonyl compounds such as aromatic aldehydes **2a**-**2b**, heterocyclic aldehydes **2c**-**2e** (furan-2-carbaldehyde **2c**, thiophene-2-carbaldehyde **2d**, and 3-formylchromone **2e**) were condensed with 5-membered active hydrogen compounds 2,4-thiazolidinedione **3a** and rhodanine **3b** in the presence of 20 mol% **1c** under ultrasound irradiations at 80°C under solvent-free conditions to provide Knoevenagel products **4**–**5** in excellent yields (Scheme 1). Aromatic aldehydes **2a**-**2b** afforded excellent yields in a shorter reaction time, whereas heterocyclic **2c**-**2e** gave high yields in a slightly long reaction time (Table 3).

**Table 3:** [**TMG**][**Lac**]-**catalyzed solvent**-**free reactions of thiazolidines 3 with aldehydes 2**

**Entry**	**Aldehydes**	**Thiazolidenes**	**Product**^**a**^	**Time (min)**	**Yield (%)**^**b**^
1	2a	3a	**4a**	20	95
2	2b	3a	**4b**	15	98
3	2c	3a	**4c**	25	91
4	2d	3a	**4d**	30	92
5	2e	3a	**4e**	20	96
6	2a	3b	**5a**	15	97
7	2b	3b	**5b**	10	99
8	2c	3b	**5c**	20	92
9	2d	3b	**5d**	25	91
10	2e	3b	**5e**	15	98

^a^Reaction conditions: **2a**-**2e** (1 mmol), **3a**-**3b** (1 mmol), and **1c** (20 mol%) were irradiated under ultrasound irradiations at 80°C. The products were characterized by spectral techniques like IR, ^1^H NMR, ^13^C NMR, and mass spectra. ^b^Isolated yields after recrystallization.

**Scheme 2:**
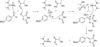
The possible reaction mechanism for the Knoevenagel reaction catalyzed by TSIL.

Active methylene compounds **3a**-**3b** afforded the Knoevenagel products selectively with exo-double bond without the formation of other side products/bis-products as shown in Scheme 2. Electron-withdrawing and electron-donating groups on aromatic aldehyde showed a slight diversion in the rate of reaction and yields, i.e., the electron-withdrawing group containing aromatic aldehydes afforded arylidene compounds **4**–**6** with better yields in a shorter reaction time (Table 3).

Next, the recyclability of the catalyst **1c** was studied using **2a** and **3a** as the model substrate. We observed that **1c** could be recovered by extraction of the filtrate with a particular solvent, and pure IL (colorless oil) was obtained after drying the water-rich layer under vacuum. The recovered catalyst was successfully recycled and reused for five runs.

As plausible reaction mechanism is concerned, **1c** catalyzed synthesis of arylidene products **4**–**5** by facilitating the nucleophilic attack of **3a**-**3b** (lactate anion abstract the active hydrogen of **3**) on the electrophilic carbon of carbonyl compounds (activated by TGA cation) **2a**-**2e**, and subsequent dehydration leads to the product formation (Scheme 2).

### Conclusion

In summary, we have disclosed a clean and efficient procedure for the synthesis of pharmacologically significant thiazolidine derivatives via Knoevenagel condensation of aldehydes with 2,4-thiazolidinedione/rhodanine. The task-specific ionic liquid [TMG][Lac] catalyst used is biodegradable, recyclable, and purely environmentally benign as it is easy to prepare, without the involvement of any harmful solvents/chemicals [31]; hence, it is better than already used imidazolium-based ionic liquids [21-25] as catalysts. The scope is fairly large as the range of aldehydes and active methylene compounds used afforded products in very good to excellent yields. Other prominent features are as follows: the reaction time is shorter, no polluting volatile solvents are used, reaction work-up is simple, no toxic by-products are formed during aqueous work-up, and all these green aspects place this method at an advantageous position compared to already reported methods for these molecules of commercial value.

### Methods

#### General

All starting materials were commercial products and were used without further purification except liquid aldehydes, which were distilled before use. Yields refer to yield of the isolated products. Melting points were determined in open capillaries in a paraffin bath and are uncorrected. Nuclear magnetic resonance spectra were obtained on a 400 MHz Bruker AMX instrument (Bruker Corporation, Billerica, MA, USA) in DMSO-d_6_ using TMS as a standard. HRMS analyses were carried out using a ESI-Q TOF instrument (Bruker Corporation). Infrared spectra were recorded using a Shimadzu FT-IR-8400 s spectrophotometer (Shimadzu Corporation, Kyoto, Japan) as KBr pellets. All the reactions were studied using a SIDILU Indian-made sonic bath (Sidilu Ultrasonic Technology, Bangalore India) working at 35 kHz (constant frequency, 120 W) maintained at 80°C without mechanical stirring.

#### General procedure for the synthesis of arylidene-thiazolidenes

A mixture of aldehyde (1 mmol), 2,4-thiazolidinedione/rhodanine (1 mmol), and [TMG][Lac] (20 mol%) was irradiated under ultrasonic irradiation at 80°C for a few minutes (see Table 3). The progress of the reaction was monitored via thin layer chromatography. After the reaction completion, the reaction mass was cooled (15°C to 20°C) and stirred with water (10 mL) for 30 min. The solid product was filtered and dried. The obtained products were recrystallized in EtOH/DMF (3:2). The products **4**–**5** were confirmed by their spectral data after comparison with authentic samples, infrared (IR), proton nuclear magnetic resonance (1H NMR), mass spectra, and melting points.

#### General procedure for the recovery of [TMG][Lac]

Further, the obtained filtrate was extracted with diethyl ether (3 × 10 mL), and the aqueous layer was dried under pressure. IL as a colorless oil was obtained which was further reused to catalyze more reactions.

#### Spectral data of reprehensive compounds

The following are the spectral data for **4a**, **4e**, **5b**, and **5e**:

•(**4a**): Mp. 249°C to 250°C, IR (KBr, cm^−1^): 3393, 1671, 1605, 1434, 1201; ^1^H NMR (300 MHz, DMSO-d_6_) *δ* 3.09 (s, 3H), 7.08 (d, 2H, *J* = 8.2 Hz), 7.52 (d, 2H, *J* = 8.2 Hz), 7.61 (s, 1H), 13.71 (s, 1H). Analysis calculated for C_11_H_9_NO_2_S_2_: C, 52.57%; H, 3.61%; N, 5.57%; S, 25.52%; found: C, 52.78%; H, 3.95%; N, 5.48%; S, 25.79%

•(**4e**) Mp.: 259°C to 260°C, IR (KBr, cm^−1^): 1647 (*γ* pyrone CO); ^1^H NMR *δ* 7.54 (ddd, 1H, 6-H), 7.62 (s, 1H, C = C-H), 7.71 (d, 1H, J_8,7_ = 8.41 Hz, 8-H), 7.98 (ddd, 1H, 7-H), 8.17 (dd, 1H, *J*_5,6_ = 8.41 Hz, *J*_5,7_ = 1.68 Hz, 5-H), 8.83 (s, 1H, 2-H), 12.41 (s, 1H, NH). Analysis calculated for C_13_H_7_NO_3_S_2_: C, 53.98%; H, 2.42%; N, 4.84%; S, 22.15%; found: C, 53.84%; H, 2.74%; N, 4.95%; S, 21.96%

•(**5b**): Mp. 268°C to 269°C. IR (KBr, cm^−1^): 3148, 1719, 1610; ^1^H NMR (300 MHz, DMSO-d_6_) *δ*_H_: 7.53 (2H, m), 7.72 (2H, m), 7.76 (1H, s), 12.65 (1H, bs). Analysis calculated for C_10_H_6_ClNO_2_S: C, 50.11%; H, 2.52%; N, 5.84%; S, 13.38%; found: C, 49.87%; H, 2.63%; N, 5.92%; S, 13.78%

•(**5e**) Mp.: 290°C, IR (KBr, cm^−1^): 1637 (*γ* pyrone CO); ^1^H NMR: *δ* 7.58 (ddd, 1H, 6-H), 7.61 (s, 1H, C = C-H), 7.74 (d, 1H, *J*_8,7_ = 8.40 Hz, 8-H), 7.88 (ddd, 1H, 7-H), 8.13 (dd, 1H, *J*_5,6_ = 8.40 Hz, *J*_5,7_ = 1.60 Hz, 5-H), 8.85 (s, 1H, 2-H), 12.48 (s, 1H, NH). Analysis calculated for C_13_H_7_NO_4_S: C 57.14%, H 2.58%, N 5.13%, S 11.73%; found: C 56.84%, H 2.74%, N 5.25%, S 11.46%

## Competing interests

The authors declare that they have no competing interests.
